# Reduced habitat quality increases intrinsic but not ecological costs of reproduction

**DOI:** 10.1002/ece3.8859

**Published:** 2022-04-19

**Authors:** Vanja T. Michel, Matthias Tschumi, Beat Naef‐Daenzer, Herbert Keil, Martin U. Grüebler

**Affiliations:** ^1^ Swiss Ornithological Institute Sempach Switzerland; ^2^ Institute of Evolutionary Biology and Environmental Studies University of Zurich Zurich Switzerland; ^3^ Forschungsgemeinschaft zur Erhaltung einheimischer Eulen e.V. Oberriexingen Germany

**Keywords:** carry‐over effects, food supplementation experiment, habitat degradation, habitat quality, life‐history trade‐off, parental care, parental costs, survival

## Abstract

Although the costs of reproduction are predicted to vary with the quality of the breeding habitat thereby affecting population dynamics and life‐history trade‐offs, empirical evidence for this pattern remains sparse and equivocal. Costs of reproduction can operate through immediate ecological mechanisms or through delayed intrinsic mechanisms. Ignoring these separate pathways might hinder the identification of costs and the understanding of their consequences. We experimentally investigated the survival costs of reproduction for adult little owls (*Athene noctua*) within a gradient of habitat quality. We supplemented food to nestlings, thereby relieving the parents’ effort for brood provisioning. We used radio‐tracking and Bayesian multistate modeling based on marked recapture and dead recovery to estimate survival rates of adult little owls across the year as a function of food supplementation and habitat characteristics. Food supplementation to nestlings during the breeding season increased parental survival not only during the breeding season but also during the rest of the year. Thus, the low survival of parents of unfed broods likely represents both, strong ecological and strong intrinsic costs of reproduction. However, while immediate ecological costs occurred also in high‐quality habitats, intrinsic costs carrying over to the post‐breeding period occurred only in low‐quality habitats. Our results suggest that immediate costs resulting from ecological mechanisms such as predation, are high also in territories of high habitat quality. Long‐term costs resulting from intrinsic trade‐offs, however, are only paid in low‐quality habitats. Consequently, differential effects of habitat quality on immediate ecological and delayed intrinsic mechanisms can mask the increase of costs of reproduction in low‐quality breeding habitats. Intrinsic costs may represent an underrated mechanism of habitat quality affecting adult survival rate thereby considerably accelerating population decline in degrading habitats. This study therefore highlights the need for a long‐term perspective to fully assess the costs of reproduction and the role of habitat quality in modifying these costs.

## Graphical abstract

With this experimental study, we show that immediate ecological costs of reproduction occur independent of habitat quality, while long‐term intrinsic costs only accumulate in habitats of low quality. Importantly, immediate costs paid during the breeding season were high, yet intrinsic costs carrying over to the post‐breeding season accumulated to a comparable magnitude (of c. 20%) over the rest of the year. Intrinsic costs may therefore represent an underrated mechanism of habitat quality affecting adult survival rate thereby considerably accelerating population decline in degrading habitats.

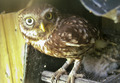

## 1 INTRODUCTION

The cost of reproduction is fundamental to the evolution of reproductive strategies and life histories and sets the basis for one of the most important life‐history trade‐offs faced by living organisms: the trade‐off between current and future reproduction. A central paradigm of life‐history theory is that parental investment into the current reproduction should reduce future reproduction, often through a reduction in parental survival (Stearns, 1992). While there is a large body of correlational and experimental studies investigating the existence and magnitude of reproductive costs in animals (e.g., Askenmo, 1979; Ghalambor & Martin, 2001; Korpimäki, 1988; Maynard Smith, 1958; Speakman, 2008), the investigation of its environmental variation and underlying mechanisms became a research priority only recently (Edward & Chapman, 2011; Pigeon et al., 2021).

Distinguishing between different mechanisms resulting in reproductive costs might be important for understanding the costs’ variation and the trade‐off's plasticity and thus the consequences for life‐history evolution in different environments. On the one hand, the importance of a distinction between intrinsic and ecological mechanisms is increasingly recognized (McNamara & Houston, 2008; Zera & Harshman, 2001). Intrinsic costs of reproduction are defined as physiological costs that arise from allocation constraints of limited resources or from the inability to simultaneously maximize all life‐history functions (Speakman, 2008). Only recently, potential physiological mechanisms of intrinsic costs were investigated in more detail (Edward & Chapman, 2011; Fowler & Williams, 2017), now even showing evidence for cumulative physiological costs over several years and reproductive bouts (Kroeger et al., 2018). In contrast, ecological costs of reproduction arise from a change in the frequency of interactions with other organisms in a way that reduces fitness (Edward & Chapman, 2011; Miller et al., 2008). Ecological costs include a higher susceptibility of reproductive individuals to predation due to courtship behavior (Tuttle & Ryan, 1981), pregnancy, egg caring, incubation (Magnhagen, 1991; Richard et al., 2017), feeding or extra foraging behavior (Ghalambor et al., 2013), or a higher susceptibility to diseases (Sheldon & Verhulst, 1996).

On the other hand, costs of reproduction have been classified into direct or indirect costs (Miller et al., 2008; Speakman, 2008). Direct costs are the pure amount of energy allocated to reproduction or, for example, the change in predation risk due to the pure extra foraging time required for reproduction. Indirect costs involve physiological trade‐offs or behavioral or physiological consequences that increase the susceptibility for predation or diseases. Thereby, costs of reproduction differ in the time scale over which they are manifested. While the intrinsic component of both the direct and indirect costs (i.e., energy/physiology), mediated through physiological trade‐offs, is most often paid after reproduction, the temporal component of the costs (i.e., time allocation to behaviors) is often immediately paid during reproduction (McNamara & Houston, 2008). As the temporal component of the costs often operates through ecological mechanisms, survival costs during reproduction are expected to be mainly due to ecological mechanisms while survival costs after reproduction are at least mediated through physiological mechanisms. That intrinsic and ecological costs often act at different time scales might be an unrecognized opportunity to discriminate between the two. However, to reach this goal, survival costs should be estimated over the course of annual routines and beyond, rather than on a year‐to‐year basis or only during reproduction.

Costs of reproduction are subject to plasticity in behavior and reproductive effort in relation to environmental conditions (Martin, 1987). For example, reduced food availability during reproduction affects both, reproductive behavior associated with predation risk (e.g., foraging behavior; Staggenborg et al., 2017) and the relative allocation of resources to reproduction and self‐maintenance (Schifferli et al., 2014). In particular, short‐lived income breeding species are expected to maintain a high current reproductive effort (Hamel et al., 2010; Martin, 1987) and often increase foraging activity under low food availability (Jacobsen et al., 2016; Staggenborg et al., 2017 but see Schifferli et al., 2014). As a consequence of this extra effort, the survival costs of reproduction should be increased when food availability is low, irrespective of whether current reproductive output is affected or not. In contrast, under high food availability the costs of reproduction may remain undetectable. Moreover, reproductive costs can be masked by individual quality (Hamel et al., 2009; Wilson & Nussey, 2010). An experimental approach is therefore necessary to disentangle the effects of environmental conditions and individual quality, by changing the parental effort while leaving the environmental conditions unchanged (Ruffino et al., 2014). Despite the fact that additional food‐dependent survival costs of reproduction are expected to affect population growth rate, only few studies have experimentally investigated the effect of habitat quality on costs of reproduction (see e.g., Barbraud & Weimerskirch, 2005; Hamel, Côté, et al., 2010; Toni et al., 2020). Therefore, demographic and evolutionary consequences of variation in natural food availability can be considerably underestimated. In the context of habitat degradation, the additional survival costs can reinforce population decline or facilitate local adaptation of reproductive life histories.

Here, we studied the effect of breeding habitat quality on survival costs of reproduction in little owls (*Athene noctua*), a short‐lived nocturnal raptor species. We experimentally supplied food to nestlings being raised in a gradient of habitat quality. Food supplementation in this species has shown to increase the nutritional state and survival of nestlings (Grüebler et al., 2018; Perrig et al., 2014), reduce traveling distance of parents for foraging trips (Jacobsen et al., 2016), and change feeding behavior of parents (Grüebler et al., 2018). Based on this evidence that reduced nestling needs resulting from extra food relax the parental workload, we predicted that food supplementation would increase parental survival and that the magnitude of this effect would depend on natural food availability. Radio‐tracking of individuals over the whole annual cycle until the next reproduction allowed us to determine the parents’ survival during both the breeding season and the post‐reproductive stages. Comparing these survival rates between parents of supplemented and un‐supplemented broods consequently allowed us to discriminate between immediate ecological and delayed physiologically mediated costs. Since short‐lived species are expected to work close to the energetic ceiling during reproduction (Hamel, Gaillard, et al., 2010), the possibility to increase parental effort in habitats with low food availability (hereafter poor habitats) might be limited and parents therefore face strong trade‐offs in the allocation of energy to reproduction and self‐maintenance. With this experimental study, we provide new insights into the intrinsic and ecological mechanisms by which food availability shapes the trade‐off between current reproduction and survival, and their potential demographic consequences.

## 2 METHODS

### 2.1 Study species and study area

**FIGURE 1:**
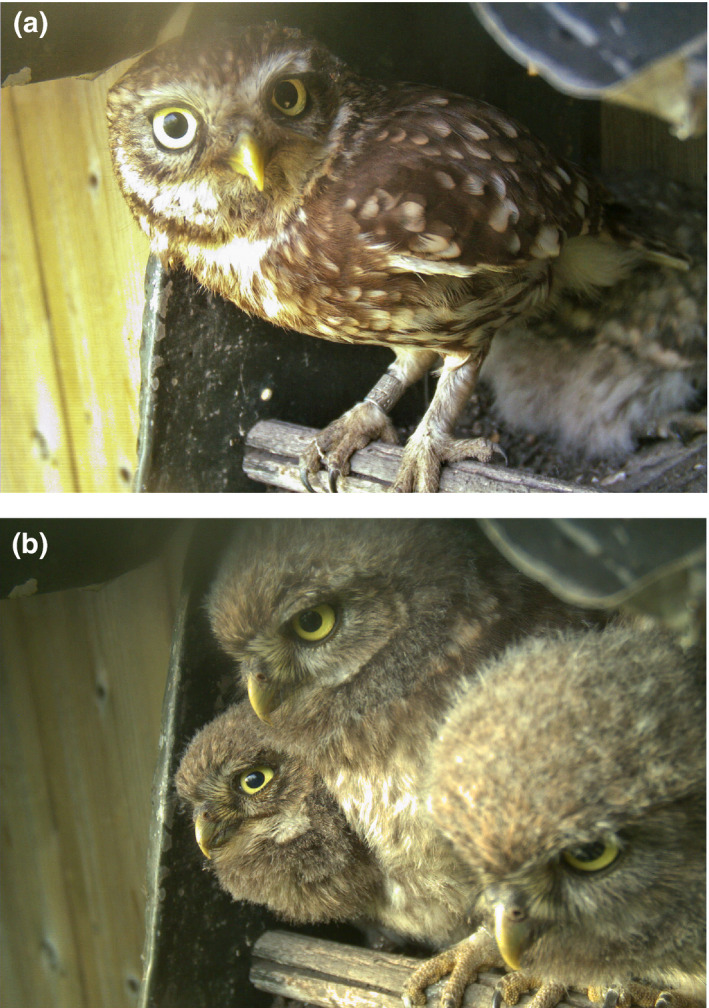
Camera trap pictures of (a) adult and (b) juvenile little owls at the nestbox. Photo credit: Swiss Ornithological Institute

The little owl *Athene noctua* (Figure 1) is a small nocturnal raptor occurring in a variety of open and semi‐open landscapes of Europe and Asia (Van Nieuwenhuyse et al., 2008). Little owls are cavity breeders and in the study area (Ludwigsburg, Baden‐Württemberg, Germany: 48°53′N, 9°11′E) mainly breed in nestboxes. Their main prey consists of small mammals, birds, arthropods, and earthworms that are caught on bare ground or grassland with low vegetation (Grüebler et al., 2018; Tschumi et al., 2020; Van Nieuwenhuyse et al., 2008). Main causes of mortality are predation and casualties associated with traffic and anthropogenic structures (Naef‐Daenzer et al., 2017). In addition, unfavorable food conditions can lead to high starvation rates (Van Nieuwenhuyse et al., 2008). Little owls are resident, territorial, and monogamous, often occupying territories for several years (Michel et al., 2017). Females are fed by males during incubation but increasingly participate in hunting and provisioning starting 5 days after hatching (Van Nieuwenhuyse et al., 2008).

The study area is characterized by intensively managed farmland interspersed with traditional tree orchards and permanent grasslands such as small, extensively managed hay meadows and pastures (mainly grazed by sheep and horses). Little owl breeding pairs occupy areas of varying quality (Michel et al., 2017), and some broods are strongly limited by food (Fattebert et al., 2018; Perrig et al., 2014). Permanent grassland and extensively cultivated orchards represent habitats with optimal food availability (Apolloni et al., 2018), and nestling survival was found to be higher in territories with an increased share of those habitats compared to territories dominated by arable land (Grüebler et al., 2018).

### 2.2 Radio tracking

From summer 2009 to summer 2012, a total of 125 adult little owls (67 females and 58 males) were caught during the early breeding season with mist nets or by removing them from nest boxes. Of those, 77 individuals (38 females and 39 males) had already been ringed as nestlings, providing us with information of their exact age. As in the long‐term ringing project in our nest box population a high rate of adult birds were already ringed, we assumed an age of one year for captures of non‐ringed birds. Each captured individual was weighed and equipped with a very high frequency (VHF) transmitter of own construction (Naef‐Daenzer et al., 2005) weighing 6.9–7.2 g (i.e., 4–5% of a bird's body mass). These transmitters have an operational range of up to 40 km and an expected life span of 400 days. Birds surviving until the subsequent breeding season were recaptured, measured again, and transmitters were replaced.

During 2–4 nocturnal visits per week, each bird was located twice at an interval of 5 min by homing in using a 3‐element Yagi antenna and a handheld receiver. In case of complete inactivity, the individual was checked for mortality the next day. Thanks to the signal of the transmitters, even buried remains could be recovered (Naef‐Daenzer & Grüebler, 2016). All recapture data were summarized into biweekly recapture histories (resulting in periods of two weeks), indicating if an individual was (1) observed alive, (2) recovered dead, or (3) neither observed alive nor recovered.

### 2.3 Food supplementation experiment

Food supplementation started when the nestlings were approximately 14 days old. We visited experimental broods (*n* = 38) and control broods (*n* = 66) every second day. Experimental broods received dead laboratory mice (deposited in the nestbox) with a total weight of 20 g per nestling during the first six visits and mice with a weight of 30 g per nestling during the subsequent 12 visits, summing to a total of 480 g additional food per nestling (Perrig et al., 2014). Control broods did not receive any supplementary food. To derive a proxy for natural food abundance close to the nest, we calculated the proportion of permanent grassland (hay fields, pastures, and orchards) within a circle of 180 m radius around each nest (i.e., an area of c. 10 ha, corresponding approximately to the home‐range size of a high‐quality little owl territory; Michel et al., 2017; Apolloni et al., 2018; Grüebler et al., 2018) using aerial images of ArcGIS 10.0 (ESRI, Redlands, CA, USA) and Google Earth (Version 7.1.2.2041, © Google 2013).

### 2.4 Statistical analyses

A mix between marked recapture and dead recovery (Kéry & Schaub, 2012; Lebreton et al., 1999) was used to model survival. This multistate model accounts for the detection probability of individuals with unknown fate (Lebreton et al., 1999). We included three true states in the model: (1) for live animals, (2) for recently dead animals, whose transmitter or remains were recovered, and (3) for recently dead but unrecovered individuals or individuals that had been dead for a while (absorbing state). During the pilot study in 2009, the development of transmitters was not finished, and consequently, the rate of transmitter loss was higher than in later years. Therefore, we included two different intercepts for the detection rate, one for 2009 and one for the other years combined. Moreover, we included a sex effect on detection probability. We assumed a constant recovery probability over all intervals. To disentangle immediate from delayed costs of reproduction, we defined two focus periods: (1) “breeding season”—defined as the period from incubation at the beginning of May until end of August, the time when juveniles leave the parental home‐range, and (2) “rest of the year”—including all remaining biweekly intervals, that is, September to April.

We then fitted the survival model including our focus variables sex, food supplementation and the amount of food‐rich habitat, as well as the interaction between food‐rich habitat and food supplementation, while controlling for the possible effects of clutch size, body mass, and age (linear and second‐level polynomial). To test whether the effects of the focus variables differed between the two time periods, we calculated the effects for each focus variable as well as the interaction between food‐rich habitat and food supplementation for the two periods separately. In addition, we included year as a fixed effect with 2010 (the first non‐pilot year) as reference year.

The amount of food‐rich habitat and clutch size were scaled to a mean of zero and a standard deviation of one prior to the analyses. Body mass was scaled to the deviation of the overall sex mean based on our data, with a value of 1 corresponding to a 10 g higher body mass than the sex‐specific mean (158.5 g for females, 152.8 g for males). Clutch size (mean ± 1 SE: experimental broods = 3.65 ± 0.14; control broods = 3.28 ± 0.17) and the amount of food‐rich habitat (mean ± 1 SE: experimental broods = 0.25 ± 0.03; control broods = 0.24 ± 0.19) did not differ between experimental broods and control broods (all *f*‐values < 0.9 and 95% credible intervals non‐overlapping zero).

All models were run in JAGS (Plummer, 2003) controlled by the R package jagsUI (Kellner, 2015) in R version 3.3.1 (R Core Team, 2015). After an adaptive phase of 10,000 iterations, three chains were run for 100,000 iterations with a burn‐in of 50,000 and no thinning. Convergence of the Markov chains was checked with Brooks–Rubin–Gelman diagnostics (Brooks & Gelman, 1998; see Methods S1 for the code of the survival model). Unless stated otherwise, model parameters and derived parameters are given as posterior means with 95% credible interval (CrI) in square brackets calculated for values of other variables set to their means and an individual age of 3 years.

## 3 RESULTS

### 3.1 Detection and recovery probability

The detection probability was lower in 2009 than in the following years (Table 1). In addition, detection probability was lower for females than males (females 2009: 0.77 [0.70 to 0.82], males 2009: 0.82 [0.75 to 0.88]; females 2010–2013: 0.91 [0.90 to 0.92], males 2010–2013: 0.94 [0.92 to 0.95]; Table 1). The transmitters of 62% [52 to 72%] of all the birds that died during the study period were recovered (Table 1).

**TABLE 1:** Model output of the biweekly survival model (*n* = 125 individuals: 67 females and 58 males)

	Posterior mean	95% CrI	*f*
Detection model			
Intercept 2009	1.189	0.862 to 1.532	
Intercept 2010–2013	2.322	2.150 to 2.502	
**Males**	**0.347**	**0.089 to 0.604**	**0.996**
Recovery probability	0.623	0.523 to 0.718	
Survival model			
Intercept BS	1.661	0.830 to 2.507	
Intercept REST	2.649	1.808 to 3.506	
**Males BS**	**0.668**	**0.053 to 1.303**	**0.984**
Males REST	0.148	−0.428 to 0.725	0.693
**Food supplementation BS**	**0.627**	**−0.154 to 1.496**	**0.939**
**Food supplementation REST**	**0.517**	**−0.157 to 1.249**	**0.931**
Food‐rich habitat BS	−0.177	−0.524 to 0.180	0.838
Food‐rich habitat REST	0.210	−0.153 to 0.603	0.864
**Food‐rich habitat × food suppl. BS**	**0.887**	**0.082 to 1.757**	**0.985**
**Food‐rich habitat × food suppl. REST**	**−0.658**	**−1.372 to 0.044**	**0.967**
**Clutch size**	**0.236**	**−0.013 to 0.491**	**0.968**
Body mass	0.064	−0.147 to 0.280	0.714
**Age**	**0.550**	**0.027 to 1.071**	**0.981**
**Age^2^**	**−0.071**	**−0.145 to 0.005**	**0.968**
Year 2009	0.026	−0.881 to 0.978	0.515
Year 2011	0.655	−0.030 to 1.331	0.969
Year 2012	0.404	−0.228 to 1.012	0.898
Year 2013	0.543	−0.398 to 1.511	0.869

Abbreviations: BS, breeding season: May–August; *f*, posterior probability, that is, proportion of the posterior distribution on the same side of zero as the mean. Variables with *f*‐values larger than 0.9 are highlighted in bold; REST, rest of the year: September–April. 95% CrI, 95% credibility intervals of posterior means.

### 3.2 Biweekly survival

**FIGURE 2:**
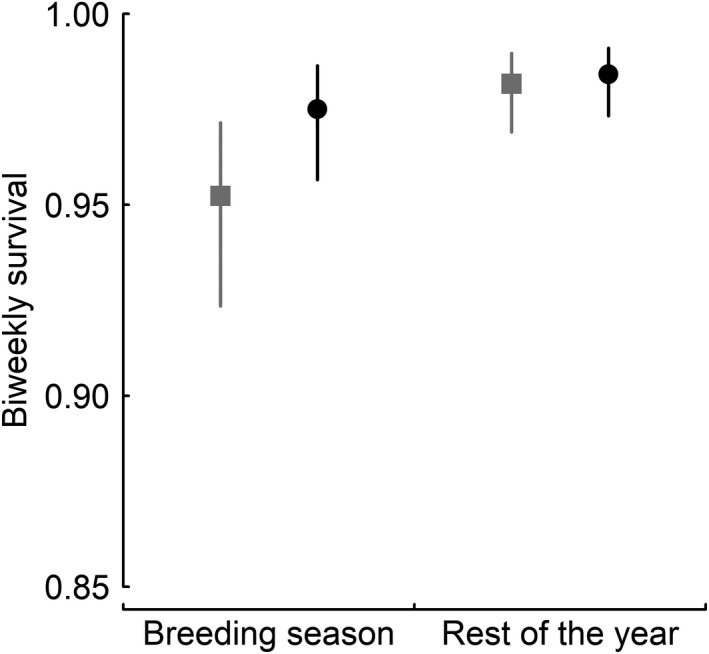
Sex‐specific survival within seasons. Biweekly survival rates of females (gray squares) and males (black circles) of un‐supplemented little owl broods during the breeding season and the rest of the year. Points represent posterior means and error bars 95% CrI. For model predictions, a mean year effect was used, individual age was set to 3 years, and all other model predictors to their mean values

For both sexes, biweekly survival of parents was lower during the breeding season than during the rest of the year (Figure 2; Table 1). In addition, female survival was considerably lower than male survival during the breeding season, whereas it was only slightly lower during the rest of the year (Figure 2; Table 1).

**FIGURE 3:**
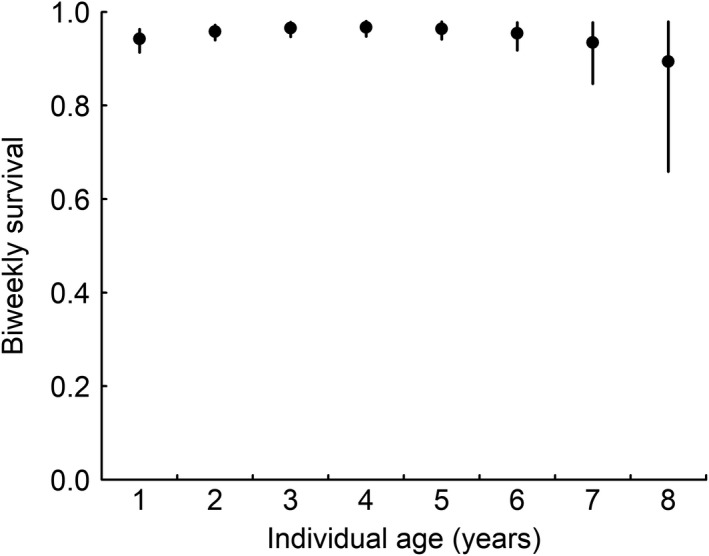
Age‐dependent biweekly survival rates of adult little owls from un‐supplemented broods during the breeding season. Points represent posterior means and error bars 95% CrI. Average year and sex effect were used for predictions and all other model predictors were set to their mean values

The age of the observed little owls ranged from one to eight years. There was a quadratic relationship between age and survival of adult little owls (Table 1, Figure 3). Thus, little owls at intermediate ages (2–6 years) had higher survival than young and old individuals (Figure 3). Furthermore, survival was positively correlated with clutch size, whereas there was little support for a relationship between survival and body mass (Table 1).

**FIGURE 4:**
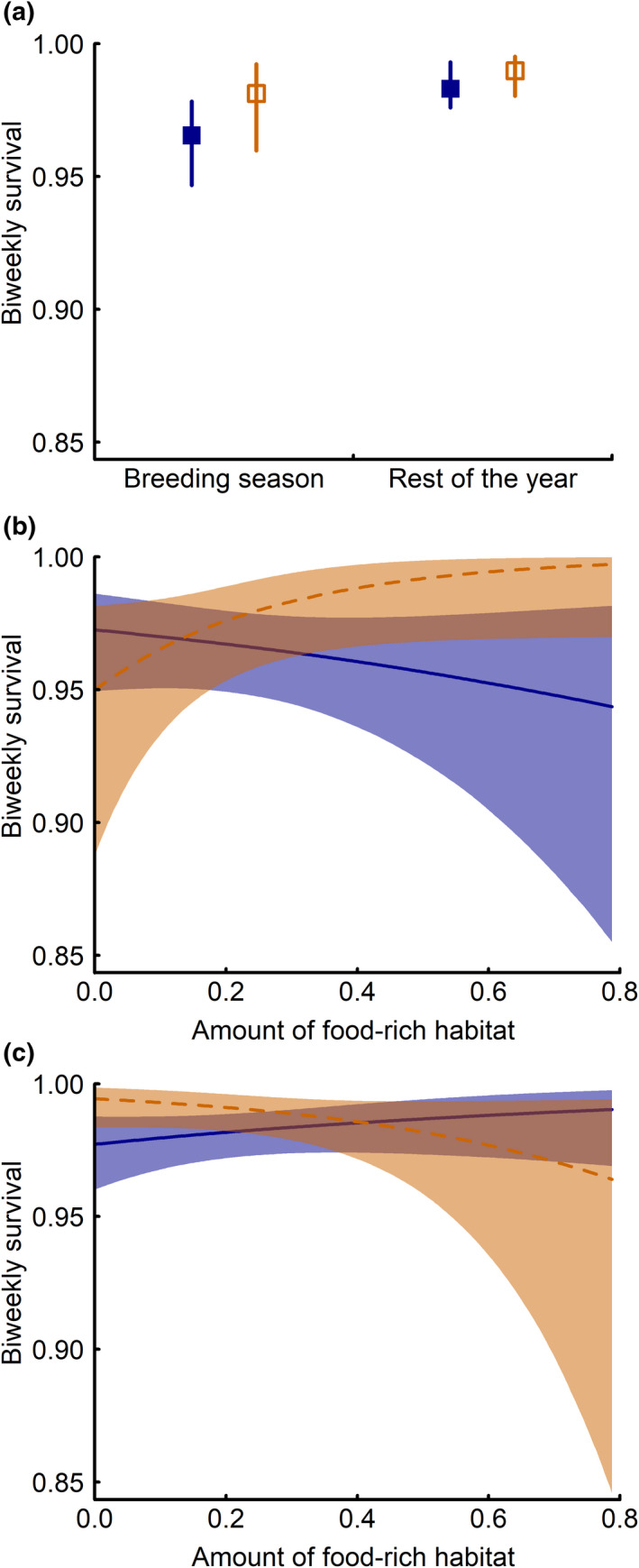
Effects of nestling food supplementation on adult survival. (a) Overall effect of supplementation: biweekly survival rates of parents of un‐supplemented (blue filled squares) and supplemented (orange empty squares) little owl broods during the breeding season and the rest of the year, (b) biweekly survival rates of parents of un‐supplemented (blue solid line) and food‐supplemented (orange dashed line) little owl broods during the breeding season in relation to the amount of food‐rich habitat around the nest, and (c) biweekly survival rates of parents of un‐supplemented (blue solid line) and food‐supplemented (orange dashed line) little owl broods during the rest of the year in relation to the amount of food‐rich habitat around the nest. Model output for the average between males and females is shown. For model predictions mean year and sex effects were used, individual age was set to 3 years, and all other model predictors to their mean values. Squares and lines represent posterior means and error bars and gray polygons 95% CrI

In general, parents of supplemented broods had higher biweekly survival rates than parents of un‐supplemented broods. This was not only true for the breeding season but also for the rest of the year (Table 1; Figure 4a). In addition, the interaction between food supplementation and the amount of food‐rich habitat in the two periods showed that in the breeding season, food supplementation enhanced survival only in territories with a high proportion of food‐rich habitat, but not in territories with a low proportion of food‐rich habitat (Table 1, Figure 4b). In contrast, during the rest of the year, food supplementation enhanced survival of adult little owls only in territories with a low proportion of food‐rich habitat (Table 1; Figure 4c).

### 3.3 Cumulative survival

**FIGURE 5:**
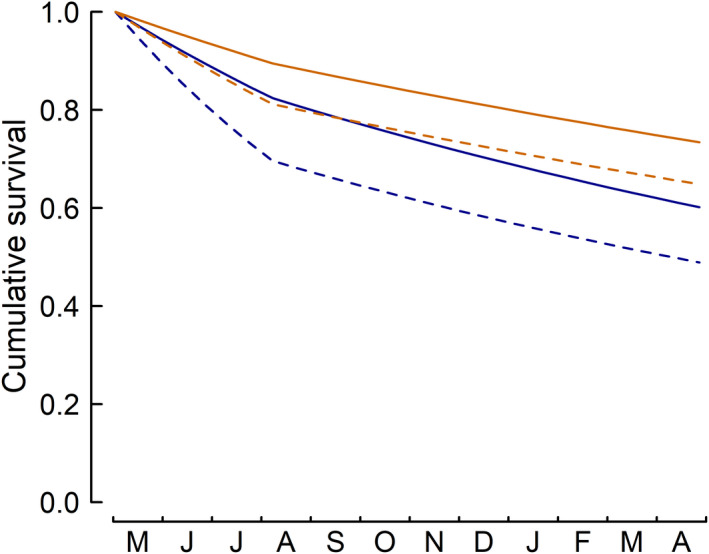
Cumulative survival of male (solid lines) and female (dashed lines) adult little owls from May to April of the subsequent year for parents of broods without food supplementation (blue) and parents of food‐supplemented broods (orange). Confidence intervals are not shown to improve readability

By multiplying the biweekly survival rates for the different periods, we estimated the cumulative seasonal and annual survival of adult little owls. Females of un‐supplemented broods had an average annual survival of 0.49 [0.34 to 0.64] and males of 0.61 [0.47 to 0.73] (Table S1; Figure 5). In contrast, females of food supplemented broods had an annual survival of 0.66 [0.48 to 0.81] and males of 0.74 [0.59 to 0.86] (Table S1; Figure 5). Thus, food supplementation of the broods increased the annual survival of both sexes considerably (females: 0.16 [0.01 to 0.31]; males: 0.13 [0.00 to 0.26]). Despite lower biweekly survival during the breeding season than during the rest of the year, cumulative survival was similar over the breeding season and the rest of the year due to the longer duration of the rest of the year (Table S1; Figure 5). While the increase in cumulative survival due to food supplementation was similar in poor (10% quantile of amount of food‐rich habitat) and food‐rich (90% quantile of amount of food‐rich habitat) habitats over the whole year, it differed considerably between seasons. In food‐rich habitats, food supplementation increased cumulative survival during the breeding season by c. 0.2 (females: 0.26 [0.06 to 0.48]; males: 0.16 [0.03 to 0.31]), whereas there was no such effect during the rest of the year (Table S1). In contrast, in poor habitats, food supplementation increased cumulative survival in the rest of the year by more than 0.2 (females: 0.24 [0.05 to 0.43]; males: 0.22 [0.04 to 0.40]), but not during the breeding season (Table S1). In addition, annual survival varied among years, with survival being lower in 2009 and 2010 compared to 2011, 2012, and 2013 (Table S2).

## 4 DISCUSSION

This study revealed distinct survival patterns of adult little owls with reduced reproductive effort (food‐supplemented broods) and control individuals across a gradient of habitat quality during the breeding season and the rest of the year. We found (1) that the biweekly survival rate of parents of un‐supplemented broods was significantly lower during the breeding season compared to the rest of the year and that this effect was particularly pronounced for females, (2) that experimental food supplementation of nestlings increased parental survival not only during the breeding season but notably also over the whole rest of the year, and (3) that the experimental effect during the breeding season was large also in high‐quality habitats, while after reproduction it was mainly present in adults breeding in low‐quality habitats. These results clearly show that environmental conditions affect the costs of reproduction. Moreover, they suggest that, irrespective of the habitat quality, the costs of reproduction are mostly paid during the time of reproduction, but that in low‐quality habitats costs also carry over to post‐reproductive phases. Our study therefore provides fundamental insights into how direct and indirect mechanisms shape survival costs of reproduction in habitats of different quality and highlights the need for a long‐term perspective to fully assess the costs of reproduction and the role of habitat quality in modifying these costs.

During the breeding season, animals are expected to mainly face direct or short‐term indirect costs mediated by ecological mechanisms (Magnhagen, 1991; Speakman, 2008). Food supplementation increases the baseline nutritional state of the nestlings and should thus allow parents to adjust their behavior in a way that reduces immediate costs (Brommer et al., 2004; Eldegard & Sonerud, 2010; Grüebler et al., 2018; Jacobsen et al., 2016; Staggenborg et al., 2017). That the biweekly survival of adults of un‐supplemented broods was lower than the survival of supplemented broods and the experimental effect stronger during the breeding season than during the rest of the year underlines that the costs of reproduction are mostly paid during the time of reproduction. As we have previously shown, the provisioning behavior of parents of un‐supplemented broods is associated with larger distances traveled and longer periods spent outside of shelters (Grüebler et al., 2018), which ultimately increases the risk of predation. Moreover, the lower survival of females compared to males during the breeding season implies that reproduction is more costly for females (Donald, 2007; Low et al., 2010). This may be mostly related to increased ecological costs such as elevated predation risks during incubation and provisioning (Magnhagen, 1991).

In contrast to immediate effects, we identified carry over effects to subsequent post‐breeding periods which are mostly related to intrinsic costs of reproduction. Presumably, parents of supplemented broods are able of building up more reserves (Brommer et al., 2004; Eldegard & Sonerud, 2010) and allocate more energy to post‐breeding life stages that in turn improve winter survival (Dawson et al., 2000; Nilsson & Svensson, 1996). The total post‐breeding increase in survival due to the food supplementation of broods in poor habitats was as high as the increase in survival during the breeding season. This suggests that both costs contribute substantially to the total survival costs of reproduction, but that the total costs of reproduction depend on the quality of the breeding habitat.

Our results show that habitat quality plays a key role in modulating the costs of reproduction. The effect of food supplementation in food‐rich habitat during the breeding season suggests that the immediate costs of reproduction are high even in territories of high quality, most probably due to predation risk during foraging. This is supported by the finding that 78% of the adult birds in this population died from predation (Naef‐Daenzer et al., 2017). In contrast, food supplementation did not increase survival in low‐quality habitats during breeding. This indicates that although habitat quality affects parental foraging behavior (Grüebler et al., 2018), the experimental treatment in poor habitats did not result in a behavioral change that allowed parents to substantially reduce ecological costs. We see three possible explanations for this pattern. First, costs of reproduction can be increased in low‐quality habitats as predicted, but the experimental approach used here was not suitable to quantify them. The amount of supplemented food was not higher in territories of low compared to high quality and may thus have been insufficient to improve conditions for parents. Second, even if supplementation allowed parents to adjust their foraging effort in poor‐quality territories, habitat configuration (low heterogeneity), and the lack of suitable hunting grounds nearby comes at the cost of increased home range sizes (Mayer et al., 2021; Michel et al., 2017), flight distances (Jacobsen et al., 2016; Staggenborg et al., 2017) and foraging trip duration (Staggenborg et al., 2017). Thus, supplementation in low‐quality territories may not relieve parents from traveling far and thereby not reduce the risk of predation. Third, food supplementation is shown to increase the number of surviving offspring more in bad than in good habitats (Grüebler et al., 2018). This may result in the extra feeding effort for a non‐reduced brood size partly absorbing the supplementation effect in poor habitats and ultimately masking the effects on survival (see also Daan et al., 1996; Eldegard & Sonerud, 2010). In conclusion, it is likely that multiple mechanisms act in concert. Yet, all three possibilities suggest that immediate ecological costs of reproduction are at least as high in low as in high‐quality habitats.

The effect of food supplementation of broods on parental survival in the post‐breeding period was only apparent in poor‐quality territories. This suggests that intrinsic physiological trade‐offs that carry over to later stages only arise in suboptimal territories. Thus, under poor, but not under favorable breeding conditions, time and energy for self‐maintenance may be strongly limited and adverse effects on the parents’ physiological state may accumulate over the breeding season.

The observed quadratic relationship of age on survival likely represents a combination of decreasing mortality with increasing experience at a younger age and increasing mortality owing to increasing senescence or higher relative investment into offspring with decreasing prospects for future reproduction at an older age (Fay et al., 2020; Tarwater & Arcese, 2017). Thereby, the peak in survival is expected to be closely related to the speed of life with species with a faster pace of life reaching this peak at a younger age than species with a slower pace of life (Jones et al., 2008).

Finally, although experimental food supplementation allows reducing parental effort irrespective of individual quality, the current design does not allow to account for the fact that high‐quality individuals are often overrepresented in high‐quality territories (Tschumi et al., 2014). High‐quality individuals could be less constrained by ecological costs due to their higher foraging effectiveness, as well as intrinsic trade‐offs, as they might be able to sustain higher reproductive effort without facing increased cost. Accordingly, the positive correlation between clutch size and survival may be linked to a positive relationship of clutch size with individual quality (Winkler & Allen, 1995). Although larger clutches require higher provisioning rates, this is often compensated by higher quality individuals for example being more efficient at foraging (Hamel et al., 2009; Wilson & Nussey, 2010). One possible interpretation of our results could therefore be that part of the interactive effects with habitat quality may represent parental quality effects. However, if clutch size is adjusted to individual quality this may actually balance the difference in costs faced by individuals of different quality, suggesting that habitat quality more likely than individual quality is causing the observed interactions.

## 5 CONCLUSIONS

We show that habitat quality differently affects ecological and intrinsic determinants of adult survival during the breeding period and during the post‐reproductive stages. During breeding, adult birds face high ecological survival costs of reproduction also in high‐quality habitats, but they only pay intrinsic post‐breeding costs when breeding in habitats of low quality. Therefore, ecological costs of reproduction during breeding can be substantial and seem to be modulated by predation pressure and environmental factors affecting predation risk. In contrast, indirect post‐breeding survival costs were mainly affected by food availability in the breeding habitat. Low habitat quality resulted in a significant reduction of adult post‐breeding survival by c. ∆*S* = 0.2. Low food availability in breeding territories thus not only affects reproductive output (Grüebler et al., 2018; Michel et al., 2017), post‐fledging survival of juveniles (Perrig et al., 2014), and adult survival during the breeding season (Oro & Furness, 2002; this study), but also post‐breeding adult survival. This unrecognized indirect effect of habitat quality on adult survival rate will add to the known negative effects of degraded habitats on demographic rates. The simultaneous increase of generalist predators and reduced food availability due to agricultural intensification is thus likely to increase both the immediate ecological and the indirect intrinsic costs of reproduction for farmland birds. To better understand the presented patterns of the costs of reproduction, future research should investigate the underlying mechanisms. Both, the environmental determinants of ecological costs of reproduction and the physiological mechanisms of intrinsic costs of reproduction, will be intriguing fields for future research.

## CONFLICT OF INTEREST

The authors declare no conflict of interest.

## AUTHOR CONTRIBUTION

**Vanja Tamian Michel:** Conceptualization (equal); Data curation (lead); Formal analysis (lead); Investigation (equal); Methodology (equal); Project administration (equal); Resources (equal); Validation (equal); Visualization (equal); Writing – original draft (equal); Writing – review & editing (equal). **Matthias Tschumi:** Conceptualization (supporting); Data curation (supporting); Formal analysis (supporting); Investigation (equal); Methodology (equal); Validation (equal); Visualization (equal); Writing – original draft (equal); Writing – review & editing (lead). **Beat Naef‐Daenzer:** Conceptualization (equal); Data curation (supporting); Formal analysis (supporting); Funding acquisition (supporting); Investigation (equal); Methodology (equal); Project administration (equal); Resources (equal); Supervision (supporting); Validation (supporting); Writing – original draft (supporting). **Herbert Keil:** Data curation (supporting); Investigation (equal); Project administration (supporting); Resources (equal). **Martin U. Grüebler:** Conceptualization (equal); Data curation (equal); Formal analysis (equal); Funding acquisition (lead); Investigation (equal); Methodology (equal); Project administration (equal); Resources (lead); Supervision (lead); Validation (equal); Visualization (equal); Writing – original draft (equal); Writing – review & editing (supporting).

## Supporting information

Supplementary Material

## Data Availability

The datasets supporting this article are available from the Zenodo vogelwarte.ch Open Repository and Archive https://doi.org/10.5281/zenodo.6421969.
