# Supplementary material for: Reduced habitat quality increases intrinsic but not ecological costs of reproduction
Source: Ecol Evol. 2022 Apr 19;12(4):e8859. doi: 10.1002/ece3.8859 (PMC9019141; doi:10.1002/ece3.8859)
Supplement: Supplementary file 1 — Supplementary Material [file ECE3-12-e8859-s001.docx]

**Supporting Information**

**Table S1.** Annual (ANN) and seasonal (BS = breeding season – May to August; REST = rest of the year – September to April) mean survival rates and 95% CrI of female and male little owls from broods with and without food supplementation, as well as increase by food supplementation in average, poor (10 % quantile of food-rich habitat) and rich (90 % quantile of food-rich habitat) territories. All other model predictors were set to their mean values.

|  | mean | 95% CrI |
| --- | --- | --- |
| **Average habitat** |  |  |
| Annual survival (ANN) |  |  |
| ANN females un-supplemented | 0.49 | 0.34 to 0.64 |
| ANN males un-supplemented | 0.61 | 0.42 to 0.73 |
| ANN females supplemented | 0.66 | 0.48 to 0.81 |
| ANN males supplemented | 0.74 | 0.59 to 0.86 |
| Increase by supplementation females | 0.16 | 0.01 to 0.31 |
| Increase by supplementation males | 0.13 | 0.00 to 0.26 |
|  |  |  |
| Seasonal survival breeding season (BS) |  |  |
| BS females un-supplemented | 0.71 | 0.57 to 0.82 |
| BS males un-supplemented | 0.83 | 0.73 to 0.91 |
| BS females supplemented | 0.82 | 0.66 to 0.93 |
| BS males supplemented | 0.90 | 0.80 to 0.96 |
| BS Increase by supplementation females | 0.11 | -0.03 to 0.26 |
| BS Increase by supplementation males | 0.07 | -0.02 to 0.16 |
|  |  |  |
| Seasonal survival rest of the year (REST) |  |  |
| REST females un-supplemented | 0.70 | 0.55 to 0.82 |
| REST males un-supplemented | 0.73 | 0.60 to 0.84 |
| REST females supplemented | 0.80 | 0.65 to 0.91 |
| REST males supplemented | 0.83 | 0.69 to 0.92 |
| REST Increase by supplementation females | 0.10 | -0.04 to 0.25 |
| REST Increase by supplementation males | 0.09 | -0.03 to 0.22 |
|  |  |  |
| **Poor habitat** |  |  |
| Annual survival (ANN) |  |  |
| ANN females un-supplemented | 0.47 | 0.30 to 0.64 |
| ANN males un-supplemented | 0.58 | 0.41 to 0.73 |
| ANN females supplemented | 0.56 | 0.32 to 0.77 |
| ANN males supplemented | 0.70 | 0.50 to 0.85 |
| Increase by supplementation females | 0.08 | -0.17 to 0.32 |
| Increase by supplementation males | 0.12 | -0.09 to 0.32 |
|  |  |  |
| Seasonal survival breeding season (BS) |  |  |
| BS females un-supplemented | 0.75 | 0.60 to 0.87 |
| BS males un-supplemented | 0.86 | 0.75 to 0.93 |
| BS females supplemented | 0.64 | 0.38 to 0.85 |
| BS males supplemented | 0.79 | 0.60 to 0.92 |
| BS Increase by supplementation females | -0.11 | -0.37 to 0.12 |
| BS Increase by supplementation males | -0.07 | -0.25 to 0.08 |
|  |  |  |
| Seasonal survival rest of the year (REST) |  |  |
| REST females un-supplemented | 0.63 | 0.45 to 0.79 |
| REST males un-supplemented | 0.67 | 0.49 to 0.82 |
| REST females supplemented | 0.87 | 0.70 to 0.96 |
| REST males supplemented | 0.89 | 0.74 to 0.97 |
| REST Increase by supplementation females | 0.24 | 0.05 to 0.43 |
| REST Increase by supplementation males | 0.22 | 0.04 to 0.40 |
|  |  |  |
| **Food rich habitat** |  |  |
| Annual survival (ANN) |  |  |
| ANN females un-supplemented | 0.49 | 0.29 to 0.68 |
| ANN males un-supplemented | 0.62 | 0.44 to 0.78 |
| ANN females supplemented | 0.61 | 0.34 to 0.83 |
| ANN males supplemented | 0.67 | 0.41 to 0.87 |
| Increase by supplementation females | 0.12 | -0.14 to 0.37 |
| Increase by supplementation males | 0.05 | -0.20 to 0.27 |
|  |  |  |
| Seasonal survival breeding season (BS) |  |  |
| BS females un-supplemented | 0.64 | 0.44 to 0.81 |
| BS males un-supplemented | 0.79 | 0.64 to 0.90 |
| BS females supplemented | 0.91 | 0.73 to 0.99 |
| BS males supplemented | 0.95 | 0.84 to 0.99 |
| BS Increase by supplementation females | 0.26 | 0.06 to 0.48 |
| BS Increase by supplementation males | 0.16 | 0.03 to 0.31 |
|  |  |  |
| Seasonal survival rest of the year (REST) |  |  |
| REST females un-supplemented | 0.75 | 0.55 to 0.89 |
| REST males un-supplemented | 0.78 | 0.61 to 0.91 |
| REST females supplemented | 0.67 | 0.40 to 0.87 |
| REST males supplemented | 0.70 | 0.44 to 0.89 |
| REST Increase by supplementation females | -0.08 | -0.35 to 0.16 |
| REST Increase by supplementation males | -0.08 | -0.33 to 0.14 |

**Table S2.** Annual survival rates [and 95% CrI] of female and male little owls with (FS) and without food supplementation across years. All other model predictors were set to their mean values.

| Year | Females | Females FS | Males | Males FS |
| --- | --- | --- | --- | --- |
| 2009 | 0.39 [0.14 to 0.66] | 0.57 [0.26 to 0.81] | 0.51 [0.25 to 0.75] | 0.664 [0.38 to 0.87] |
| 2010 | 0.38 [0.16 to 0.62] | 0.56 [0.30 to 0.78] | 0.50 [0.27 to 0.72] | 0.661 [0.43 to 0.84] |
| 2011 | 0.60 [0.41 to 0.76] | 0.74 [0.57 to 0.87] | 0.70 [0.54 to 0.82] | 0.806 [0.67 to 0.90] |
| 2012 | 0.52 [0.34 to 0.69] | 0.68 [0.49 to 0.83] | 0.63 [0.47 to 0.77] | 0.760 [0.61 to 0.88] |
| 2013 | 0.56 [0.31 to 0.78] | 0.70 [0.45 to 0.88] | 0.66 [0.44 to 0.84] | 0.779 [0.57 to 0.92] |

**Methods S1.** BUGS code of the biweekly survival model

model {

# -------------------------------------------------

# Parameters:

# s: true survival probability

# r: recovery probability

# p: detection probability

# -------------------------------------------------

# States (S):

# 1 alive in study area

# 2 recently dead and recovered

# 3 recently dead, but not recovered, or dead (absorbing)

# Observations (O):

# 1 seen alive

# 2 recovered dead

# 3 neither seen nor recovered

# -------------------------------------------------

# Define state-transition and observation matrices

for (i in 1:nind){

# Define probabilities of state S(t+1) given S(t)

for (t in f[i]:(l[i]-1)){

ps[1,i,t,1] <- s[i,t]

ps[1,i,t,2] <- (1-s[i,t])*r[i,t]

ps[1,i,t,3] <- (1-s[i,t])*(1-r[i,t])

ps[2,i,t,1] <- 0

ps[2,i,t,2] <- 0

ps[2,i,t,3] <- 1

ps[3,i,t,1] <- 0

ps[3,i,t,2] <- 0

ps[3,i,t,3] <- 1

} # t

for (t in f[i]:l[i]){

# Define probabilities of O(t) given S(t)

po[1,i,t,1] <- p[i,t]

po[1,i,t,2] <- 0

po[1,i,t,3] <- 1-p[i,t]

po[2,i,t,1] <- 0

po[2,i,t,2] <- 1

po[2,i,t,3] <- 0

po[3,i,t,1] <- 0

po[3,i,t,2] <- 0

po[3,i,t,3] <- 1

} #t

} #i

# Likelihood

for (i in 1:nind){

for (t in (f[i]+1):l[i]){

# State process: draw S(t) given S(t-1)

z[i,t] ~ dcat(ps[z[i,t-1], i, t-1,])

} #t

for (t in f[i]:l[i]){

# Observation process: draw O(t) given S(t)

y[i,t] ~ dcat(po[z[i,t], i, t,])

} #t

} #i

# Priors and constraints

for(i in 1:nind){

for (t in f[i]:(l[i]-1)){

logit(s[i,t]) <- a.s[period[t]] +

beta.males.period.s[period[t]] * male[i] +

beta.food.sup.s[period[t]] * food.sup[i,index.year[t]] +

beta.prop.green.s[period[t]] * prop.green.s[i,t] +

beta.prop.green.food.sup.s[period[t]] * prop.green.s[i,t] *

food.sup[i,index.year[t]] +

beta.clutch.size.s * clutch.size.s[i,index.year[t]] +

beta.bodymass.s * bodymass.s[i,index.year[t]] +

beta.age.s * age[i,index.year[t]] +

beta.age.sq.s * age.squared[i,index.year[t]] +

beta.year.s[index.year[t]]

r[i,t] <- mean.r

} #t

for (t in f[i]:l[i]){

logit(p[i,t]) <- a.p[period2[t]] + beta.males.p * period3[t] * male[i]

} #t

} #i

# Priors for survival

for(i in 1:2){

a.s[i] ~ dnorm(0, 0.44)

beta.males.period.s[i] ~ dnorm(0, 0.44)

beta.prop.green.s ~ dnorm(0, 0.44)

beta.food.sup.s[i] ~ dnorm(0, 0.44)

beta.prop.green.food.sup.s[i] ~ dnorm(0, 0.44)

}

beta.clutch.size.s ~ dnorm(0, 0.44)

beta.bodymass.s ~ dnorm(0, 0.44)

beta.age.s ~ dnorm(0, 0.44)

beta.age.sq.s ~ dnorm(0, 0.44)

# Priors for recovery

mean.r ~ dunif(0, 1)

# Priors for detection probability

for(i in 1:2){

a.p[i] ~ dnorm(0, 0.44)}

beta.males.p ~ dnorm(0, 0.44)

beta.year.s[1] ~ dnorm(0, 0.44)

beta.year.s[2] <- 0

beta.year.s[3] ~ dnorm(0, 0.44)

beta.year.s[4] ~ dnorm(0, 0.44)

beta.year.s[5] ~ dnorm(0, 0.44)

} #model
